# A Randomized Steady-State Bioavailability Study of Synthetic *versus* Natural (Kiwifruit-Derived) Vitamin C

**DOI:** 10.3390/nu5093684

**Published:** 2013-09-17

**Authors:** Anitra C. Carr, Stephanie M. Bozonet, Juliet M. Pullar, Jeremy W. Simcock, Margreet C. M. Vissers

**Affiliations:** 1 Centre for Free Radical Research, Department of Pathology & Biomedical Science, University of Otago, Christchurch, PO Box 4345, Christchurch 8140, New Zealand; E-Mails: stephanie.bozonet@otago.ac.nz (S.M.B.); juliet.pullar@otago.ac.nz (J.M.P.); margreet.vissers@otag (M.C.M.V.); 2 Department of Plastic and Reconstructive Surgery, University of Otago, Christchurch, PO Box 4345, Christchurch 8140, New Zealand; E-Mail: jeremy.simcock@cdhb.health.nz

**Keywords:** ascorbate, ascorbic acid, human, plasma, urine, semen, leukocytes, skeletal muscle

## Abstract

Whether vitamin C from wholefoods has equivalent bioavailability to a purified supplement remains unclear. We have previously showed that kiwifruit provided significantly higher serum and tissue ascorbate levels than synthetic vitamin C in a genetically vitamin C-deficient mouse model, suggesting a synergistic activity of the whole fruit. To determine if these results are translatable to humans, we carried out a randomized human study comparing the bioavailability of vitamin C from kiwifruit with that of a vitamin C tablet of equivalent dosage. Thirty-six young non-smoking adult males were randomized to receive either half a gold kiwifruit (*Actinidia Chinensis* var. *Hort 16A*) per day or a comparable vitamin C dose (50 mg) in a chewable tablet for six weeks. Ascorbate was monitored weekly in fasting venous blood and in urine, semen, leukocytes, and skeletal muscle (*vastus lateralis*) pre- and post-intervention. Dietary intake of vitamin C was monitored using seven day food and beverage records. Participant ascorbate levels increased in plasma (*P* < 0.001), urine (*P* < 0.05), mononuclear cells (*P* < 0.01), neutrophils (*P* < 0.01) and muscle tissue (*P* < 0.001) post intervention. There were no significant differences in vitamin C bioavailability between the two intervention groups in any of the fluid, cell or tissue samples tested. Overall, our study showed comparable bioavailability of synthetic and kiwifruit-derived vitamin C.

## 1. Introduction

Vitamin C (ascorbate) is an essential water-soluble micronutrient that is obtained through the diet primarily from fruits and vegetables [1]. The bioavailability of dietary vitamin C represents the proportion absorbed by the intestines and available for metabolic processes within the body. Vitamin C is actively transported into the body via two sodium-dependent vitamin C transporters, SVCT1 and SVCT2, which exhibit different tissue distributions and uptake kinetics [2,3]. SVCT1 is expressed in epithelial tissue and is primarily responsible for intestinal uptake and renal reabsorption of vitamin C, the latter maintaining whole body homeostasis [3]. SVCT2 is expressed in specialized and metabolically active tissues and is required for delivery of vitamin C to tissues with a high demand for the vitamin either for enzymatic reactions [4] and/or to help protect these tissues from oxidative stress [3].

Kiwifruit are rich in vitamin C [5] and we have previously used a genetically vitamin C-deficient mouse model (the *Gulo* mouse) to investigate the comparative bioavailability of synthetic *versus* kiwifruit-derived vitamin C [6]. Interestingly, we found that kiwifruit gel provided higher serum, leukocyte, heart, liver and kidney levels of ascorbate than equivalent amounts of purified vitamin C, suggesting a synergistic activity of the whole fruit in this model. Although synthetic and food-derived vitamin C are chemically identical, the bioavailability of vitamin C could potentially be affected by the numerous micronutrients and phytochemicals with antioxidant properties that are present in fruits and vegetables [7,8]. For example, kiwifruit contain reasonable amounts of vitamin E [9], which has been shown to spare vitamin C in an animal model [10]. Kiwifruit also contain numerous different flavonoids [9,11], some of which can inhibit the *in vitro* oxidation of vitamin C via direct scavenging of free radicals and/or chelation of redox-active metal ions [7,8].

To determine if the results of our animal study [6] are translatable to humans, we carried out a randomized human study comparing the bioavailability of vitamin C from gold kiwifruit (*Actinidia Chinensis* var. *Hort 16A*) with a tablet of equivalent dosage. We have previously shown that consumption of half a gold kiwifruit per day results in a significant increase in plasma ascorbate in individuals with low initial levels (<23 µmol/L) [12]. For this study, we chose a dose of half a kiwifruit per day and the equivalent 50 mg/day vitamin C since this dose lies on the steeply rising portion of the sigmoidal plasma bioavailability curve [13]. This enhances the likelihood of detecting a difference between the two interventions compared with doses >100 mg/day where plasma saturation is approached [13].

Several previous studies have tested the comparative bioavailability of synthetic *versus* food-derived vitamin C utilizing plasma and/or urine levels [14,15,16,17,18]. However, only one has investigated the comparative bioavailability of vitamin C in leukocytes [19]. Therefore, in addition to plasma and urine, we have monitored the bioavailability of vitamin C in peripheral blood mononuclear cells and neutrophils, seminal fluid and skeletal muscle tissue [20] before and after the six week intervention. We also monitored the participants’ dietary intake of vitamin C using seven day food and beverage records.

## 2. Study Design and Methods

### 2.1. Participants

This study was conducted according to the guidelines laid down in the Declaration of Helsinki and all procedures involving human participants were approved by the Upper South Regional Ethics Committee (#URA/11/02/003). The study was registered with the Australian New Zealand Clinical Trials Registry (#ACTRN12611000162910).

Non-smoking males aged 18–35 years from local tertiary institutes were screened to ascertain their eligibility for the study. Exclusion criteria included recent smoker (within previous year), allergy/intolerance to kiwifruit, taking vitamin C-containing supplements (within past three months), taking prescription medication (within past three months), excessive alcohol consumption (>21 standard drinks/week), high fruit and vegetable consumption (>5 servings per day), diabetes mellitus, bleeding disorders, and fainting due to fear of needles. Anthropometric measurements were carried out to determine body mass index (BMI) and a fasting venous blood sample was drawn to determine plasma ascorbate levels as described below.

Sample size calculations indicated that at 80% power and alpha = 0.05, a sample size of 15 participants per intervention group would detect a minimum difference of 10 µmol/L ascorbate as determined using data derived from our previous vitamin C bioavailability study [12]. To allow for potential withdrawal during the study, 36 non-smoking participants (18 per group) with below average plasma ascorbate levels were enrolled for the study and provided written informed consent.

### 2.2. Study Design

**Figure 1:**
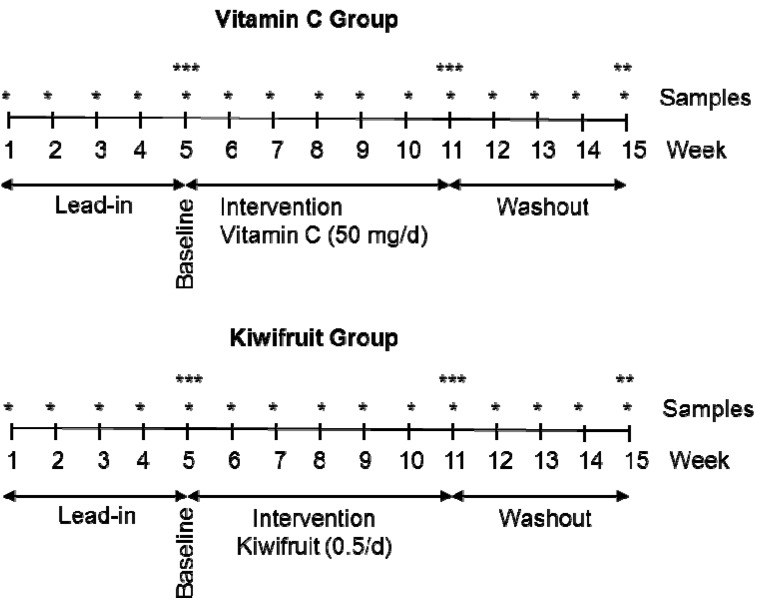
Parallel groups study design. * Weekly plasma samples; ** urine, semen and leukocyte samples; *** urine, semen, leukocyte and skeletal muscle samples.

The study employed a parallel arms design and the participants were randomized into a 50 mg vitamin C per day group or a half kiwifruit per day group using a random numbers chart. A parallel arms rather than cross-over study design was chosen to avoid potential confounding by kiwifruit-derived constituents, e.g., vitamin E, which may not wash out prior to the vitamin C supplement phase of a cross-over study. A lead-in phase of five weeks allowed the participants time to control their dietary vitamin C intake by eliminating juice and substituting high vitamin C foods, e.g., citrus and kiwifruit, with low vitamin C foods, e.g., apples and bananas (guidelines were provided as to the vitamin C content of common foods). This was followed by an intervention phase of six weeks and a washout phase of four weeks (Figure 1). Fasting venous blood samples were drawn weekly to monitor plasma ascorbate levels. Twenty four hour urine, semen, and leukocyte samples were collected at week five (baseline), week 11 (post-intervention) and week 15 (post-washout). Muscle biopsies were carried out at baseline and post-intervention. Participants also completed four seven-day food and beverage records (at the beginning of the study, pre- and post-intervention and post-washout).

### 2.3. Interventions

Chewable orange-flavored vitamin C tablets were provided by Tishcon Corp., Westbury, NY, USA. Analysis of the tablets indicated that they contained 52 mg of vitamin C per tablet. Participants in the vitamin C tablet arm of the study were asked to consume one tablet per day.

Gold kiwifruit (*Actinidia chinensis* var. *Hort. 16A*) were provided by Zespri International Ltd., Mount Maunganui, New Zealand, and were stored at ≤4 °C. The vitamin C content of the kiwifruit was monitored by HPLC with electrochemical detection [12] and indicated that the kiwifruit flesh contained 116 ± 10 mg vitamin C per 100 g (mean ± SD, *n* = 5); this value did not change during cold storage of the fruit. Participants in the kiwifruit arm were asked to consume half a fresh kiwifruit daily, not including the skin. Therefore, the actual amount of vitamin C consumed was estimated to be ~53 mg per half a kiwifruit.

### 2.4. Sample Collection and Processing

#### 2.4.1. Plasma, Urine and Semen

Peripheral blood was collected into 5 mL K_3_-EDTA vacutainer tubes and plasma was isolated by centrifugation at 4 °C. Urine was collected over 24 h into pre-weighed collection bottles containing K_2_-EDTA. Semen was collected into pre-weighed collection containers containing K_2_-EDTA and kept cold until processed. The plasma, urine and semen samples were extracted with perchloric acid containing DTPA, as described previously [12], prior to storage at −80 °C until HPLC analysis.

#### 2.4.2. Mononuclear Leukocytes and Neutrophils

White blood cells were purified from heparinized whole blood as described previously [20], the cell pellets counted and extracted with perchloric acid containing DTPA, and the supernatants stored at −80 °C until HPLC analysis.

#### 2.4.3. Skeletal Muscle Tissue

A small piece of tissue (~14 mg) was removed from the *vastus lateralis* using a Quick-Core biopsy needle (14 gauge, 6 cm long with a 20 mm throw, from Cook Medical Inc., Bloomington, IN, USA), and processed as described previously [20], extracted with perchloric acid containing DTPA, and stored at −80 °C until HPLC analysis.

### 2.5. Analysis of Vitamin C by HPLC

The ascorbate content of the kiwifruit, plasma, urine, semen, leukocytes and muscle tissue was analyzed using reverse-phase HPLC with electrochemical detection as described previously [12]. Briefly, samples were separated on a Synergi 4 µ Hydro-RP 80A 150 × 4.6 mm column (Phenomenex NZ Ltd., Auckland, New Zealand) using a Waters 600 solvent delivery system with a Hitachi L-2200 refrigerated autosampler and an ESA Coulochem II electrochemical detector (+200 mV electrode potential and 20 µA sensitivity). The mobile phase comprised 80 mM sodium acetate buffer, pH 4.8, containing DTPA (0.54 mmol/L) and freshly added paired-ion reagent *n*-octylamine (1 µmol/L), delivered at a flow rate of 1.2 mL/min. A standard curve of sodium-l-ascorbate, standardized spectrophotometrically, was freshly prepared for each HPLC run in 77 mmol/L HPLC-grade perchloric acid containing DTPA (100 µmol/L).

### 2.6. Analysis of Food and Beverage Records

The vitamin C content of the food and beverage dietary records was estimated using *Diet Cruncher* software [21], and the New Zealand FOODfiles Food Composition Database (2006) as described previously [12].

### 2.7. Statistical Analysis

Data is represented as mean ± SD for group characteristics and mean ± SEM for comparison of group means. The differences between paired and unpaired data were determined by two-tailed Students’s *t-*tests and *P* values ≤ 0.05 were considered significant. Analysis of variance with Fisher pairwise multiple comparison procedure was carried out using *SigmaStat* software [22].

## 3. Results

### 3.1. Participant Characteristics

One hundred and thirty four non-smoking individuals were screened and their fasting plasma ascorbate concentrations determined. The average ± SD plasma ascorbate concentration for these individuals was 48 ± 16 µmol/L (Table 1). Thirty six of the individuals with below average plasma ascorbate levels, who also satisfied the other inclusion criteria, were enrolled in the study and randomized into the vitamin C tablet group or the half kiwifruit per day group. The average ± SD fasting plasma ascorbate levels of the enrolled groups were 31 ± 11 µM and 34 ± 10 µM, with a range of 3 µM to 44 µM (Table 1). There were no significant differences between the two groups.

**Table 1:** Characteristics of individuals screened and enrolled in the study.

	Screened ^a^ (*n* = 134)	Vitamin C Group ^a^ (*n* = 18)	Kiwifruit Group ^a^ (*n* = 18)
Age (years)	21 ± 3	21 ± 3	22 ± 4
Weight (kg)	81 ± 16	84 ± 19	89 ± 23
Height (cm)	182 ± 7	181 ± 7	181 ± 7
BMI (kg/m^2^)	24 ± 4	26 ± 5	27 ± 6 *
Ascorbate (µmol/L)	48 ± 16	31 ± 11 **	34 ± 10 **

^a^ Data represent mean ± SD; * *P* < 0.05, ** *P* < 0.001 for unpaired *t*-test of intervention groups *versus* screened group. There were no significant differences between the intervention groups.

### 3.2. Dietary Intake of Vitamin C

Analysis of the food and beverage records indicated a vitamin C intake of ~30 mg/day at baseline (Table 2). Addition of the vitamin C tablet or half a kiwifruit per day to the daily diet of the two groups resulted in an increase in vitamin C intake, with both groups approaching ~75 mg (Table 2), and no difference between the two groups (*P* = 0.512). Following four weeks washout, the vitamin C intake had returned to baseline levels.

**Table 2:** Change in vitamin C intake and ascorbate concentration in body fluids, cells and tissue following supplementation with 50 mg vitamin C or half a kiwifruit per day for six weeks.

	Vitamin C group ^a^ (50 mg/day)			Kiwifruit group ^a^ (half/day)			Between group
	**Baseline**	**Intervention**	**Washout**	**Baseline**	**Intervention**	**Washout**	**Intervention** *P* **value** ^b^
Intake (mg/day)	31.2 ± 3.1	76.7 ± 2.6 ***	29.1 ± 2.9	28.6 ± 3.1	73.4 ± 4.2 ***	31.4 ± 5.1	0.512
Plasma (µmol/L)	23.5 ± 2.5	51.3 ± 3.5 ***	34.3 ± 4.4 *	22.7 ± 2.5	45.5 ± 2.5 ***	30.5 ± 3.1	0.860
Urine (µmol/24 h)	42.8 ± 9.2	104.2 ± 27.6 *	59.5 ± 19.9	32.3 ± 8.1	70.5 ± 17.0 *	64.7 ± 29.8	0.503
Semen (µmol/L)	284.8 ±27.0	321.0 ± 33.3	264.4 ± 31.1 *	326.3 ± 47.5	378.8 ± 42.7	237.7 ± 19.6 *	0.676
Mononuclear cells (nmol/10^8^ cells)	38.7 ± 6.2	84.5 ± 6.3 ***	78.5 ± 4.3 **	60.5 ± 6.1	90.9 ± 6.1 **	75.2 ± 5.2	0.227
Neutrophils (nmol/10^8^ cells)	21.9 ± 3.1	39.6 ± 3.8 **	24.9 ± 2.1	13.7 ± 2.4	30.4 ± 2.5 ***	24.8 ± 1.7 ***	0.798
Skeletal muscle (nmol/g)	14.5 ± 2.0	61.3 ± 3.5 ***	nd	15.1 ± 2.5	52.8 ± 5.0 ***	nd	0.429

^a^ Data represent mean ± SEM; ^b^ *P* values were determined by unpaired *t*-test of vitamin C group *versus* kiwifruit group post-intervention following subtraction of baseline values; * *P* < 0.05 and ** *P* < 0.01 and *** *P* < 0.001 for paired *t*-test of intervention *versus* baseline. nd = not determined.

### 3.3. Vitamin C Status of Plasma, Urine and Semen

**Figure 2:**
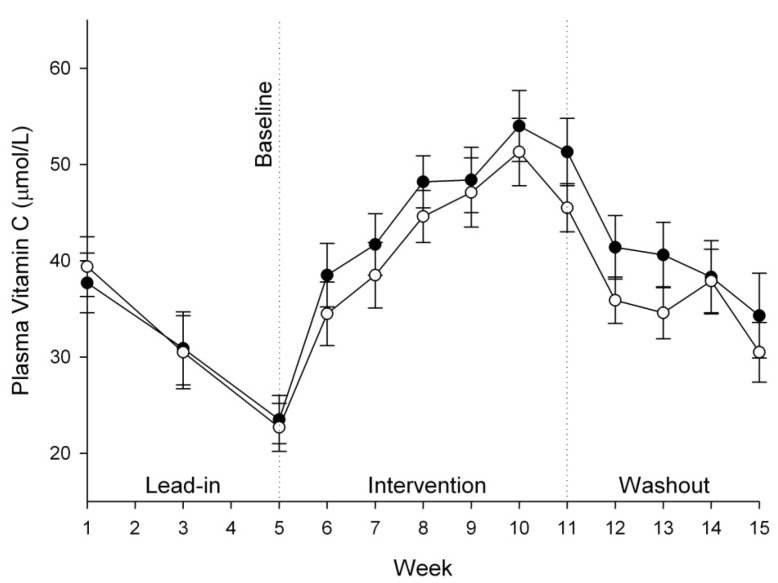
Plasma ascorbate concentrations in vitamin C group (50 mg/day, ●) and kiwifruit group (0.5/day, ○). Data represent mean ± SEM. Two way analysis of variance with Fisher pairwise multiple comparison procedure indicated a significant increase in plasma ascorbate from one week post-intervention (week 6) onwards, but no significant difference between the two interventions.

Plasma ascorbate levels increased significantly during the six week intervention phase, from ~23 µmol/L to a maximum of ~50 µmol/L (Table 2). There were no significant differences between the two groups during this period or during the four week washout phase (Figure 2). Similarly, there were no significant differences between the two treatment groups for either urinary ascorbate excretion (*P* = 0.503) or seminal ascorbate levels (*P* = 0.676) (Table 2).

### 3.4. Vitamin C Status of Leukocytes and Skeletal Muscle Tissue

The ascorbate levels in mononuclear cells and neutrophils in both intervention groups increased following the six week intervention (Table 2), but there were no differences between the two interventions (*P* = 0.798). The baseline skeletal muscle tissue ascorbate levels were ~15 nmoL/g wet weight, and following intervention there were 3.5- to 4-fold increases in tissue ascorbate levels in both treatment groups (Table 2). Once again, there were no significant differences between the two intervention groups (*P* = 0.429).

## 4. Discussion

In direct contrast to our previous *Gulo* knockout mouse study, which showed clear differences in comparative vitamin C bioavailability [6], this investigation has shown no differences in the steady-state bioavailability of synthetic *versus* kiwifruit-derived vitamin C in plasma, semen, peripheral blood leukocytes and skeletal muscle of humans. Other comparative bioavailability studies in guinea pigs have shown similar results to our animal study [23,24,25,26,27]. Enhanced uptake of vitamin C into specific organs of guinea pigs (e.g., adrenals and spleen) was observed when administered with flavonoid-rich juices/extracts or purified plant flavonoids [23,24,25,26,27], although comparable vitamin C accumulation was observed in some organs (e.g., liver) in several studies [23,24,25,28], suggesting either tissue specific differences or effects of differences in study design.

In agreement with our current human study, others have shown little difference in steady-state plasma and/or urine bioavailability of synthetic vitamin C and that found in different fruits, fruit juices and vegetables [14,15,16,17,18,19]. Only one previous study has investigated the comparative bioavailability of synthetic *versus* natural vitamin C in leukocytes [19]. Although neutrophils express SVCT2 [29], when their respiratory burst is activated they primarily transport the oxidized form of ascorbate (dehydroascorbic acid) via the glucose transporters GLUT 1 and GLUT 3, followed by intracellular reduction [30]. In support of our observations with neutrophils and mononuclear cells, Pelletier *et al.* [19] found no difference in leukocyte ascorbate uptake between synthetic vitamin C (in the presence or absence of rutin) and that from orange juice. Interestingly, an *in vitro* study showed that the flavonoids myricetin and quercitin inhibited the uptake of both ascorbate and dehydroascorbic acid into monocytic (HL-60 and U937) and lymphocytic (Jurkat) cells [31]. Whether this occurs *in vivo* is, however, uncertain due to the low plasma bioavailability of flavonoids [32].

Although leukocyte ascorbate status is often used as an indicator of whole body status, whether this is an accurate model for other tissues and organs is uncertain. This premise is supported by our animal study which indicated that different organs exhibited maximal uptake at varying doses of vitamin C [6]. Therefore, we also investigated the previously unreported effects of synthetic *versus* natural vitamin C on skeletal muscle ascorbate status. Ascorbate is transported into muscle cells via SVCT2 [33,34]. In our study muscle tissue exhibited a greater relative uptake of ascorbate than leukocytes, however, there was again no difference in bioavailability between the synthetic and fruit-derived vitamin C.

We also investigated seminal fluid ascorbate status. Although the baseline seminal fluid ascorbate was lower than vitamin C replete and healthy non-smoking men [35,36,37], little change was observed with the low dose vitamin C tablet or half kiwifruit per day dose. Early studies have shown that vitamin C intakes of up to 250 mg/day are required to return depleted seminal ascorbate to normal levels [35,36,38], indicating that higher intakes of vitamin C, e.g., those that result in plasma saturation, are required to increase seminal fluid levels [39].

## 5. Conclusions

Numerous animal studies have shown differences between the tissue bioavailability of synthetic and natural or flavonoid-rich vitamin C. However, more evidence is accumulating to indicate that this is not the case in humans. Our current human study showed no difference in bioavailability to plasma, semen, peripheral blood leukocytes and skeletal muscle of kiwifruit-derived vitamin C compared with a chewable vitamin C tablet of equivalent dosage. Thus, other nutrients and phytochemicals present in kiwifruit appear to be neither enhancing nor inhibiting the uptake of vitamin C from the whole fruit. However, vitamin C is known to enhance the bioavailability of other nutrients, such as non-heme iron [40,41], and kiwifruit also contain numerous micronutrients and phytochemicals which will undoubtedly confer considerable health benefits in addition to the positive effects of the high levels of vitamin C also delivered by this fruit [42].
